# Assessing Readiness of International Investigations into Alleged Biological Weapons Use

**DOI:** 10.3201/eid3107.240841

**Published:** 2025-07

**Authors:** Maximilian Brackmann, Anja Blasse, Júlio Gouveia Carvalho, Cindi R. Corbett, Cédric Invernizzi, Una Jakob, Stefan Kloth, Filippa Lentzos, Ines Mergler, Per Wikström

**Affiliations:** Spiez Laboratory, Spiez, Switzerland (M. Brackmann, C. Invernizzi); Robert Koch Institute, Berlin, Germany (A. Blasse, S. Kloth, I. Mergler); Biological Defense Laboratory, Lisbon, Portugal (J.G. Carvalho); National Microbiology Laboratory, Winnipeg, Manitoba, Canada (C.R. Corbett); Peace Research Institute Frankfurt, Frankfurt, Germany (U. Jakob); King’s College London, London, UK (F. Lentzos); Swedish Defense Research Agency FOI, Umea, Sweden (P. Wikström).

**Keywords:** bioterrorism and preparedness, Outbreak investigation, public health, biological weapons, united nations, secretary-general, UNSGM

## Abstract

Without clarity if an outbreak is natural, accidental, or deliberate, infectious disease outbreaks of unknown or ambiguous origin can lead to speculation of a purposeful biological attack. Outbreaks in conflict settings are particularly prone to suspicions and allegations. In an increasingly confrontative global geopolitical landscape and with active information manipulation, outbreaks of ambiguous origin are likely to increase concerns of the deliberate use of biological agents. The United Nations General Assembly has agreed on and the United Nations Security Council has endorsed a mechanism to investigate allegations of deliberate use titled the United Nations Secretary-General’s Mechanism for Investigation of Alleged Use of Chemical or Biological Weapons. A recent full-scale field exercise evaluated the deployment readiness of the mechanism and found it is well placed to investigate suspicious disease outbreaks, with room for continual improvement.

Unusual disease outbreaks are increasingly likely to raise suspicions about their origins, particularly during conflicts, when they might be accompanied by misinformation and active disinformation (*1*,*2*). Recent examples include the outbreak of Legionnaires’ disease in Poland in a city serving as a hub for humanitarian and military aid to Ukraine (*1*,*2*) and the outbreak of Ebola virus disease in Uganda (*3*). Those outbreaks were accompanied by suspicions of deliberate release (*1*–*3*). Investigations into the source of disease outbreaks are typically conducted by local health authorities and inform measures taken to prevent further spread. In the Legionnaires’ disease and Ebola virus disease outbreaks, careful and thorough investigations by local public health agencies revealed their natural origin (*3*–*7*).

If findings suggest the possibility of a deliberate origin, with indicators such as multiple geographically dispersed index cases with high sequence similarity of the infecting agent, unusually high lethality rates, atypical manifestations in humans or animals, an unusual infecting agent for the location or time of year, or suspicious circumstances linked to the outbreak, then a national law enforcement investigation might be initiated (*8*). To corroborate the investigation, especially in a conflict setting or in locations where political tensions are high, an international investigation might also be called for (*9*).

## Investigating Disease Outbreaks with Unclear Origins

The use of a biological agent as a weapon or to deliberately cause harm is explicitly prohibited by the 1925 Geneva Protocol and implicitly prohibited by the 1972 Biological and Toxin Weapons Convention (BWC), which also bans development, production, acquisition, transfer, and stockpiling of biological weapons (*10*–*12*). However, those treaties do not currently provide a mandate to investigate allegations of biological weapons use. The text of the BWC only provides the option to lodge a complaint with the United Nations (UN) Security Council (UNSC) about possible breaches of the convention. There is no mechanism in place for the UNSC to act in response to such a complaint. To date, Russia is the only country that has called for an investigation into a potential violation of the BWC through the UNSC, invoking Article VI of the convention in 2022. The UNSC did not accept the request for an investigation into the alleged violation. Independent of the UNSC, in which its composition, procedures, and political dynamics could make agreeing on an investigation difficult, an alternative investigation mechanism does exist. The UN Secretary-General’s Mechanism for Investigation of Alleged Use of Chemical and Biological Weapons (UNSGM) was established by the UN General Assembly in 1987 and recognized by the UNSC in 1988 (*13**–**15*). The UNSGM was established after reports emerged of the use of chemical weapons during the 1980–1988 Iran–Iraq War.

The UN Office for Disarmament Affairs (UNODA) serves as the custodian of the UNSGM and maintains rosters of experts and laboratories the UN Secretary-General can consult and rely on when the mechanism is activated. All UN member states can nominate experts and laboratories to those rosters. The mission team would be selected, depending on the event particularities, from the roster of qualified experts. The various skills needed within the team depend on the circumstances of the mission but in most cases include interviewing and sampling skills to collect evidence. A further roster of expert consultants is available to advise the Secretary-General on many different aspects of the investigation, such as the credibility of the allegations, the composition of the mission team, or the feasibility of an on-site investigation. Expert consultants are high level specialists recognized in their fields of expertise. In addition, a network of rostered analytical laboratories is available to receive and analyze samples taken by a mission team (*13*).

Any UN member state can bring an instance of alleged use of chemical, toxin, or biological weapons to the attention of the Secretary-General and request an investigation. The Secretary-General then decides whether to launch a mission on the basis of the information submitted by the member state, and “… only in extraordinary circumstances would the Secretary-General not carry out an investigation at the site of the alleged incident…” (*16*). The countries concerned are expected to accept the investigation and cooperate with the mission team. The Secretary-General can request the advice of expert consultants on the report of alleged use of biological weapons “… and to develop continuously the measures required for the smooth conduct of the investigations” (*16*).

Once the Secretary-General deems an investigation is warranted, a team of qualified experts would conduct the investigation, likely including interviews and the collection of samples. Identical sets of samples would be sent to >2 rostered laboratories for analysis. Results of laboratory analyses would be reported to the mission team, which would then compile and present a final report to the Secretary-General, who communicates the results to all UN member states.

To date, the UNSGM has been activated 3 times, in each instance to investigate the alleged use of chemical weapons. The first investigation mission to Mozambique and the second to Azerbaijan were conducted in 1992. The Mozambique investigation was inconclusive, and the investigation in Azerbaijan did not find evidence of chemical weapon use (*17*). The third mission was conducted in 2013 in Syria with mission team members from several UN member states, the World Health Organization, and the Organization for the Prohibition of Chemical Weapons. The mission concluded that chemical weapons were used in 4 of the 7 instances under investigation (*17*).

The principles of the investigation mechanism are described in the guidelines and procedures of the UNSGM and their annexes (*18*). Those guidelines encourage UN member states to nominate experts to advise the Secretary-General (expert consultants) and those staffing a possible mission team (qualified experts). The guidelines also call for nominations of analytical laboratories (*16*). In all 3 categories, the western European and others group have nominated the most, followed by Asia and the Pacific region. Latin America and the Caribbean have the fewest nominations (*16*). Geographic representation on the mission team is necessary for the political credibility of investigation findings, and equitable representation of UN regions on the rosters remains a priority. In addition to nominations, the UNSGM Guidelines and Procedures asks member states to provide trainings and exercises for experts and laboratories.

## Trainings and Exercises

Many countries support a range of activities to strengthen the UNSGM (*17*), such as providing quality assurance exercises for rostered laboratories (*19*). Other activities include developing training modules or guidance documents and hosting basic training or advanced skills courses for rostered experts. A structured training program for nominated qualified experts has been in place since 2017, including a basic training course laying the foundation and training the basics of the mechanism. After completing the basic training course, participants receive training on safe and secure approaches in field environments and specialized trainings on aspects of a mission. The training courses encompass small practical modules with limited complexity to focus on specific aspects of an investigation mission. The basic training course includes a 2–3-day scenario-based exercise, in which participants can put the training into practice.

Larger-scale field exercises provide opportunities to simulate the entire investigation process of a mission, including mission planning; negotiations with local governments and relevant institutions; interviews with affected persons, medical staff, and other stakeholders; field sampling; laboratory identification and characterization of the agent; and reporting and media handling. Those large-scale field, or capstone, exercises are particularly valuable to test the alignment of different trainings with the requirements of a mission and to highlight potential areas to grow expertise.

## The 2020 and 2022 Capstone Exercise

In September 2020, Germany hosted a predeployment tabletop exercise, held virtually because of COVID-19 travel restrictions. After that exercise, a 10-day full-scale field exercise in Berlin occurred 2 years later, in September 2022 (*20*). The simulated mission team consisted of 19 rostered experts from 16 countries selected by UNODA on the basis of their expertise and previous participation in basic training courses for the UNSGM. Proficiency of the qualified experts in English was necessary for the smooth conduct of the exercise and is likely also necessary for a real-world mission. Representatives from UNODA, the UN Internal Task Force, analytical laboratories, and expert consultants also participated in the exercise. This capstone exercise was the second time such an exercise was conducted for the UNSGM; the first was held in 2014.

The exercise scenario was set in a fictional UN member state with an outbreak of *Yersinia pestis* that was resistant to many antimicrobial drugs and cases of pneumonic and bubonic plague with human-to-human transmission that mainly occurred in refugees. In the scenario, German was the primary language spoken in the affected country, although many interviewees, border officials, and other country officials were proficient in English to varying degrees. The team was provided with interpreters for interviews with non–English-speaking interview partners. The affected country suspected a biological weapons attack on its refugee reception facilities by a neighboring country, with which it had a politically strained relationship. The affected country requested the UN Secretary-General to conduct a UNSGM investigation.

**Figure 1:**
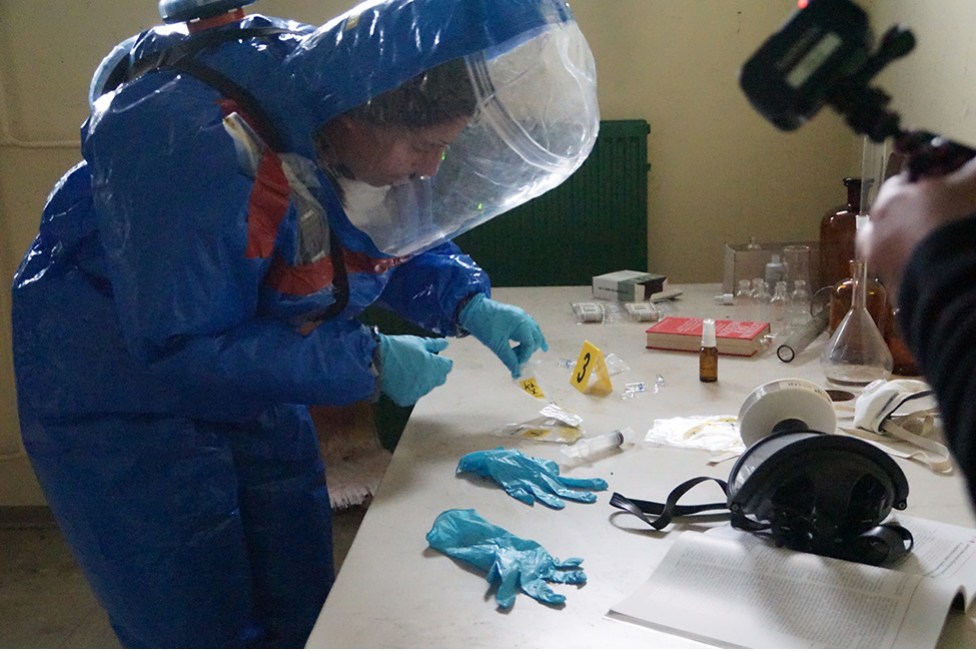
Environmental sampling of a mission team member during the 2020 and 2022 capstone exercises to strengthen international investigations of alleged biological weapons use. Photograph taken by Bernd Bruckmoser.

During the artificial deployment with the aim to conduct interviews and take samples (Figure 1), the mission team was confronted with challenging border crossings, host-country negotiations, media pressure, a delicate security situation, and high political tensions. Some of the situations were exacerbated by language barriers, which the team handled by using team members as translators. After the investigation was concluded, the mission head presented the findings in an interim report to fictional representatives of the UN.

The exercise was accompanied by an evaluation team of 10 observers, coordinated by the Swedish Defense Research Agency FOI, who summarized their observations in an evaluation report published in June 2023 (*21*). The authors of this manuscript were part of either the organizing or evaluation teams.

## Key Lessons

The capstone exercise demonstrated the operational readiness of the UNSGM to be called on to investigate an allegation of biological weapons use. The exercise also demonstrated the training program for rostered experts was successful. The simulated mission team had a good understanding of the investigative process and of the UNSGM.

**Figure 2:**
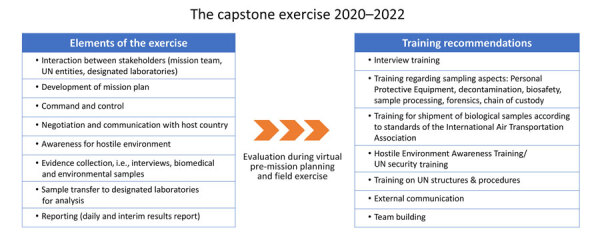
Design of the exercise scenario and learnings and recommendations deduced from the 2020 and 2022 capstone exercises to strengthen international investigations of alleged biological weapons use. UN, United Nations.

Field exercises are used and designed to demonstrate where there is room for continual improvement. In the capstone exercise, those key areas were identified (Figure 2). The importance of planning, both in terms of mission planning and operations planning, as well as adhering to a clear command-and-control plan and the need for familiarity with the technical equipment used, were highlighted by the exercise. The UNSGM does not list or provide a universally agreed-upon set of equipment required for a UNSGM mission because of the specialized nature of the equipment required. Specific, fit-for-purpose equipment should be procured before each mission. The UN, member states, international organizations, or the private sector can provide any required equipment outside the standard UN equipment. 

A large part of mission planning can be conducted before the mission starts, or even before a mission is requested, by having procedures and agreements in place for selecting the mission team members, having arrangements for equipment, and team members being aware of their roles and responsibilities. After the capstone exercise, efforts were made to address the issue of deployment timeliness with a workshop held by UNODA and VERTIC, which developed a predeployment package for qualified experts. Such preparations could shorten the time needed to prepare an investigation. A mission team is supposed to be dispatched within 48 hours after the UN Secretary-General decides to launch an investigation, and a timely investigation of a suspected biological attack increases the chances of obtaining conclusive results. 

For sampling, the exercise illustrated the necessity of clarifying sampling site and sample prioritization and visual information recording. Because of the exercise, a dedicated course on sampling was developed. The exercise highlighted learnings related to sample processing, sample transfer, chain-of-custody, and analysis, which is reflected in the development of both the sampling course and a course in which qualified experts can obtain the required certification for shipment of infectious substances and toxins by the International Air Transport Association. 

Any investigation of an alleged biological weapons incident will rest not only on biomedical and environmental samples but also on information gained from interviews. The capstone exercise highlighted the need to strengthen data-gathering from interviews and to train experts in the specialized interviewing skills required for such a particular investigation, which led to the establishment of a course on investigative interviewing. 

Both participants and observers found that a clearer picture of the supportive and coordinative functions of the UN Internal Task Force would aid rostered experts in their roles. The importance of external communication was emphasized, and the evaluation team suggested additional training on when and how individual mission team members can practically and psychologically deal with social media pressure targeting the mission team or individual mission team members.

## Conclusion

In the currently tense geopolitical climate, unusual disease outbreaks might be increasingly accompanied by information manipulation and disinformation campaigns, particularly in conflict zones. Suspicions and allegations about biological weapons are likely to become more frequent. Trends to this effect are already visible, such as in the allegations of BWC noncompliance and in disinformation campaigns on prohibited biological weapons-related activities, which formed a topic of formal consultations at the UN in Geneva, Switzerland, in 2022 (*22**–**24*). Allegations of actual use of biological weapons would have even more serious repercussions. A well-supported and well-functioning UNSGM, which can independently gather the evidence to confirm or refute allegations, is therefore in the interest of all UN member states. Robust investigation capabilities are also in the interest of national authorities, and in particular public health authorities, whose credibility could be undermined by a bioweapons-related disinformation campaign. A concurrent advantage for member states to contribute to and participate in national activities that strengthen the UNSGM is to provide participants with relevant insights and considerations for investigating potential deliberate events. Such specialized insights and considerations are unique and can offer useful perspectives for national outbreak investigations.

Training and exercises to strengthen the UNSGM are solely funded through member states, and continued support is crucial for a well-resourced and rapidly deployable investigation team. Although the time between the first and the second capstone exercise was 8 years, future large-scale field exercises could be conducted more frequently. Field exercises combined with an augmented training schedule will support faster adaptation and evolution of training schemes and give more qualified experts the opportunity to test and refine their skills, contributing to a well-trained, regionally diverse roster of qualified experts willing and ready to offer their services to the UNSGM.
